# An Atypical Pediatric Headache: Outpatient Presentation of Petrous Apicitis With Cerebral Venous Sinus Thrombosis

**DOI:** 10.1155/crpe/3216868

**Published:** 2026-03-11

**Authors:** Merrick J. Harris, Paola Pedraza Cruz, Janet Moore, Deepa Mukundan

**Affiliations:** ^1^ Department of Pediatrics, The University of Toledo College of Medicine and Life Sciences, Toledo, Ohio, USA, utoledo.edu; ^2^ Department of Pediatrics, The University of Toledo Medical Center, Toledo, Ohio, USA, utoledo.edu; ^3^ Division of Infectious Diseases, Nationwide Children’s Hospital, Toledo, Ohio, USA, nationwidechildrens.org

## Abstract

**Background:**

Severe complications of acute otitis media (AOM) are rare in the United States due to widespread antibiotic use. One severe complication of AOM is the contiguous spread of infection to the petrous apex of the temporal bone, causing petrous apicitis. Due to its proximity to the dural venous sinuses, petrous apicitis can lead to a septic cerebral venous sinus thrombosis (CVST). The presentation of these complications is discussed here in the case of an 8‐year‐old male.

**Case Report:**

An 8‐year‐old male with no significant past medical history presented to an outpatient clinic with a history of intermittent headaches. Examination revealed bilateral papilledema, swelling behind the right ear, and a right middle ear effusion, resulting in a prompt referral to the emergency department. Imaging demonstrated mastoiditis, petrous apicitis, and thrombosis of the right sigmoid and transverse sinuses extending into the internal jugular vein. He was admitted to the pediatric intensive care unit and treated with anticoagulation, empiric antibiotics, and acetazolamide for intracranial hypertension. His course was complicated by the development of abducens neuropathy and somnolence, and he underwent right mastoidectomy with sigmoid sinus decompression. Intraoperative cultures grew *Streptococcus intermedius*. Postoperatively, he improved clinically, though persistent papilledema and abducens nerve palsy were noted. He was discharged after a ten‐day inpatient stay on a regimen of oral antibiotics, rivaroxaban, and acetazolamide, with multidisciplinary follow‐up.

**Conclusions:**

Petrous apicitis with septic CVST is a rare but life‐threatening complication of AOM. This case emphasizes the need for vigilance in children presenting with severe or persistent neurologic symptoms in the setting of prior otologic symptoms. Prompt recognition and treatment are critical to reducing morbidity and preventing long‐term neurologic sequelae.

## 1. Introduction

Severe complications of acute otitis media (AOM) are uncommon in the United States due to widespread treatment with antibiotics [1]. Petrous apicitis is a severe complication of AOM involving infection of the petrous apex of the temporal bone, occurring in an estimated 1 in 50,000 children with AOM, and is associated with high morbidity [1, 2]. Rarely, a septic cerebral venous sinus thrombosis (CVST) may occur secondarily due to the proximity of the petrous apex to the dural venous sinuses [3]. When this occurs, prompt treatment using a combination of antibiotics, anticoagulation, and surgical intervention may be necessary to treat the nidus of infection and prevent cranial nerve deficits, intracranial hemorrhage, seizure, and other dangerous complications [4]. Here, we discuss the case of an 8‐year‐old previously healthy male who developed petrous apicitis and a septic CVST and his initial presentation with these conditions in the outpatient setting. This case highlights the importance of early recognition of rare complications of otogenic infections and demonstrates the value of a multidisciplinary treatment approach to prevent lasting neurologic sequelae.

## 2. Case Presentation

An 8‐year‐old male with no significant past medical history and up‐to‐date immunizations presented to an outpatient pediatric clinic with a 1‐week history of intermittent right‐sided headaches. Two days prior, the patient had experienced a severe headache associated with diplopia. During this episode, the patient experienced excruciating right‐sided head pain and emergency medical services (EMS) were called by the parents, but upon their examination, the patient was determined to be stable and was not transported to the hospital. He was recommended to follow up with his pediatrician as an outpatient. At the time of this outpatient visit 2 days later, the patient was completely asymptomatic.

Upon questioning, approximately 2 months prior, the patient had developed cold‐like symptoms and right ear pain and was evaluated at an urgent care facility. At this visit, a right middle ear effusion was noted, but an AOM episode was ruled out. Loratadine was prescribed at this time under the assumption that allergies were contributing to his symptoms, and his symptoms seemed to resolve. Subsequently, he developed a transient fever and was noted to be lethargic for a week by his mother, with one episode of emesis. He began experiencing headaches intermittently after this, which occasionally woke him from sleep but were not associated with morning emesis. Diplopia was noted only during the most recent severe headache episode for which EMS was called.

**FIGURE 1:**
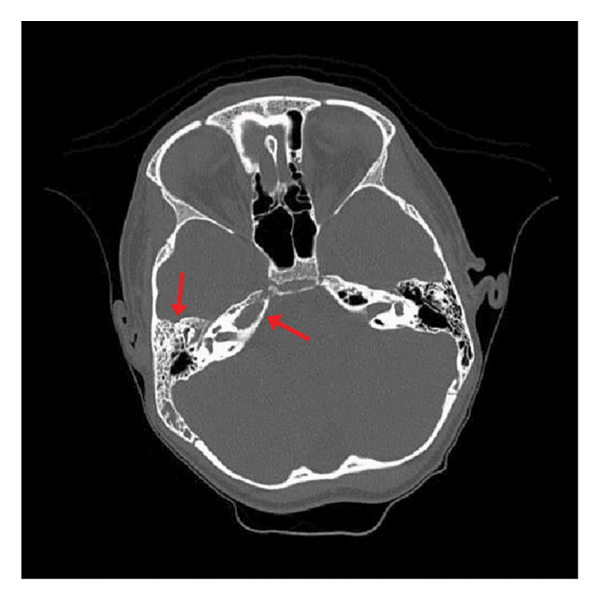
CT temporal bone on admission demonstrating right mastoid and middle ear opacities, predominantly within the epitympanum, with mild thinning of the tegmen tympani and tegmen mastoideum. No discrete ossicular erosion was observed. Pneumatization of the bilateral petrous apices with opacification of the right petrous apex is shown.

On physical examination, swelling was noted behind the right tympanic membrane and a right middle ear effusion. Ophthalmoscopy revealed bilateral papilledema, and the patient was referred to the emergency department (ED) for a suspected complicated otogenic infection. At the ED, the patient experienced another severe right‐sided headache associated with photophobia and phonophobia. A noncontrast CT scan was obtained and was negative for any acute intracranial trauma or hemorrhage, and a CT temporal bone revealed right mastoid and middle ear opacification (Figure 1). The patient received a migraine cocktail and was admitted for further observation.

**FIGURE 2:**
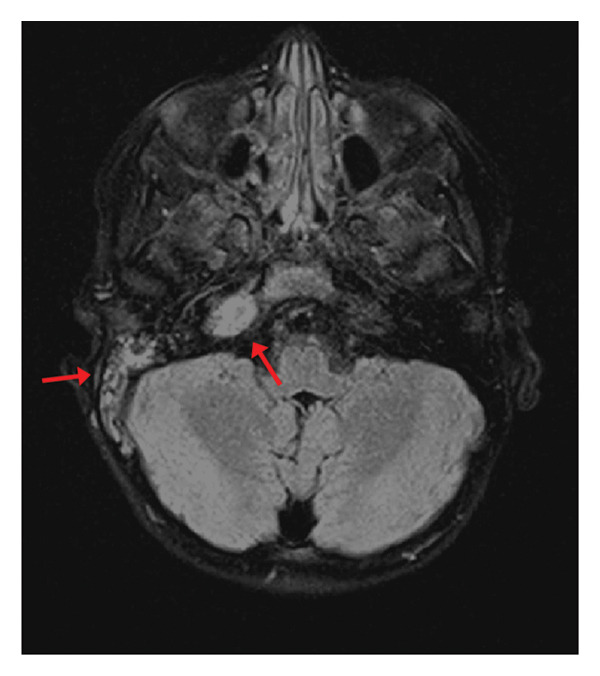
MRI brain on admission demonstrating right mastoid enhancement and notable coalescent purulent contents within the petrous apex, concerning for right mastoiditis and right petrous apicitis. No pathologic brain parenchymal signal or enhancement and no convincing evidence for meningitis were seen.

**FIGURE 3:**
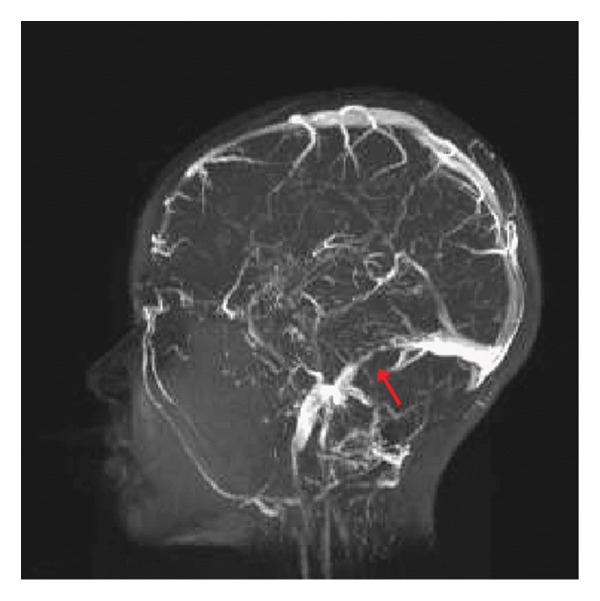
MRV on admission demonstrating thrombus in the right sigmoid and distal transverse sinuses extending to the proximal internal jugular vein; a presumed infected thrombus.

An MRI of the brain with and without contrast was obtained (Figure 2), which revealed right mastoid enhancement and notable coalescent purulent contents within the petrous apex, concerning for right mastoiditis and right petrous apicitis. A thrombosis was discovered involving the right sigmoid and distal transverse sinuses, extending to the proximal portion of the internal jugular vein, visualized on MRV (Figure 3). The patient was immediately transferred to the pediatric intensive care unit (PICU) for elevated care.

At the PICU, the patient was started on anticoagulation and empiric antibiotic therapy for a presumed septic thrombus. Neurosurgery was consulted and deferred intervention, and otolaryngology recommended a right mastoidectomy initially, but deferred surgery after a repeat CT scan showed improvement from this treatment regimen.

On Hospital Day 2, the patient underwent a right myringotomy with tympanostomy tube placement. Intraoperative aspirate of the middle ear fluid was cultured and grew *Streptococcus intermedius*. The patient was resumed on anticoagulation after surgery and started on acetazolamide for elevated intracranial pressure (ICP). Blood cultures remained negative throughout his hospital stay.

The patient reported improvement in symptoms but experienced persistent diplopia and new‐onset somnolence on Hospital Day 7. Repeat imaging revealed partial resolution of the thrombosis but persistent mastoid and petrous inflammation. Given his worsening neurologic status, the patient was emergently transferred to a tertiary pediatric facility.

At the tertiary center, bilateral papilledema and left abducens nerve palsy were noted on examination. On Day 1 post‐transfer, the patient underwent a right mastoidectomy with sigmoid sinus decompression and tympanostomy tube replacement. Significant purulent fluid and granulation tissue were noted intraoperatively. His antibiotic regimen was narrowed to IV ampicillin and eventually to a 14‐day course of oral amoxicillin. Anticoagulation was transitioned to rivaroxaban. Acetazolamide was continued and increased to 1250 mg daily due to persistent papilledema. The patient had no further headaches postoperatively, although he reported a new‐onset tingling sensation in his extremities. Ophthalmologic exam revealed stable 2+ papilledema and a mild left abduction deficit, for which he adopted a head turn to compensate for visual deficits. Inflammatory markers (CRP and ESR) improved, and the patient was discharged from the hospital after a total of 10‐day inpatient stay. Ongoing follow‐up was arranged with specialists in pediatric neurology, infectious disease, hematology, ophthalmology, and otolaryngology.

**FIGURE 4:**
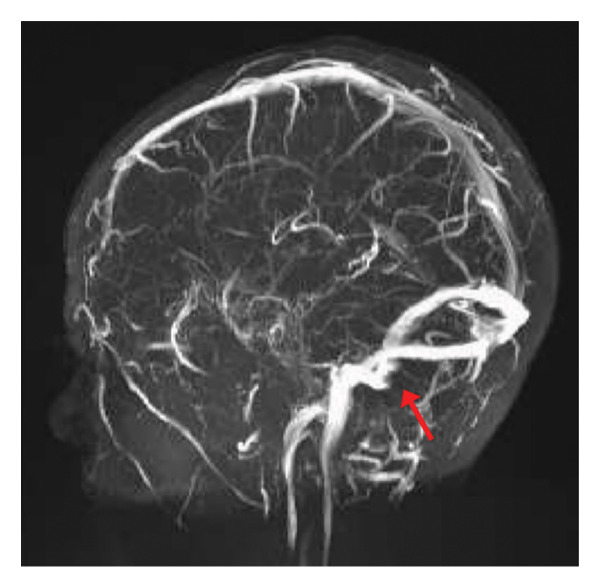
MRV of the brain at 5 months postdischarge demonstrating an improvement in the residual chronic thrombus within the distal transverse and right sigmoid sinuses. The proximal right internal jugular vein is patent.

**FIGURE 5:**
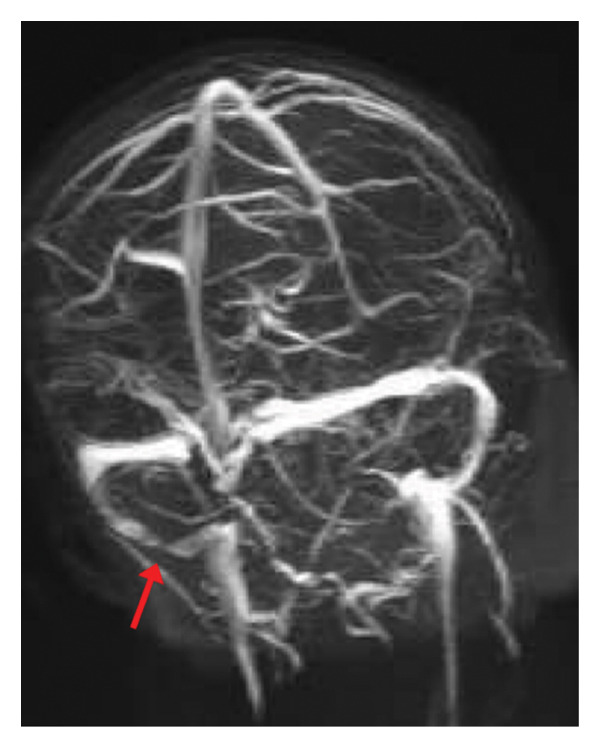
Alternate view of the MRV of the brain at 5 months postdischarge. Moderate stenosis of the distal right sigmoid sinus is observed and may be related to postsurgical changes and scar formation from the septic thrombus and sigmoid sinus decompression.

As an outpatient, the patient was continued on rivaroxaban 15 mg daily and acetazolamide 1250 mg daily. At 2 months postdischarge, a repeat MRV demonstrated persistent stenosis of the distal right sigmoid sinus. After 5 months postdischarge, another repeat MRV of the brain was performed, this time demonstrating an improvement in the residual chronic thrombus within the distal transverse and right sigmoid sinuses (Figures 4 and 5). The proximal right internal jugular vein was found to be patent.

At this point, acetazolamide was tapered and discontinued as intracranial pressures were stabilized, and there was no recurrence of papilledema or cranial nerve VI palsy. Rivaroxaban was continued by hematology until it was deemed safe to discontinue at approximately 8 months postdischarge, with no changes in additional imaging.

## 3. Discussion

This case illustrates a rare progression from an episode of AOM to petrous apicitis with septic CVST in a pediatric patient. Petrous apicitis and CVST are extremely rare complications of AOM in children [1]. In this case, the organism *Streptococcus intermedius* was implicated and is known for its propensity to form abscesses and invasive infections [5]. A member of the *Streptococcus anginosus* group, this organism is associated with increased rates of intracranial complications, empyema formation, and overall morbidity in otitis media and sinusitis cases in pediatric patients [5]. A more commonly implicated organism in septic CVSTs is *Fusobacterium necrophorum,* the agent frequently causing Lemierre’s syndrome, which is characterized by septic thrombophlebitis of the internal jugular vein following an oropharyngeal infection [6]. Similar to *Streptococcus intermedius, Fusobacterium necrophorum* is described to trigger clot formation through direct bacterial invasion of vasculature, vascular inflammation, and platelet activation [6]. Neurologic complications of petrous apicitis include cranial neuropathies, Gradenigo syndrome, meningitis, and encephalitis [2]. CVST causes intracranial hypertension due to impaired venous drainage and results in a range of clinical symptoms, including papilledema, cranial nerve VI palsy, seizure, venous infarction, and intracranial hemorrhage [7]. In this patient, persistently elevated ICP with bilateral papilledema, abducens neuropathy, and altered mental status was observed. Management of these conditions requires a multidisciplinary approach involving prompt neurosurgical consultation, antimicrobial therapy, anticoagulation, and ICP‐lowering agents such as acetazolamide [4, 7]. Regular monitoring of visual function is also necessary to prevent persistent visual deficits [4, 7]. This patient’s initial asymptomatic presentation in the outpatient setting distracted from his severe underlying pathology, which was only suspected after a thorough history and physical examination. This case reinforces the importance of reassessment in pediatric patients with persistent or worsening neurologic symptoms, especially those with a history of ear complaints suggestive of an episode of AOM.

Petrous apicitis and septic CVST are extremely rare but life‐threatening complications of AOM in children. This case underscores the importance of early recognition, imaging, and aggressive multidisciplinary management of these conditions. Clinicians should maintain a high index of suspicion in pediatric patients with prolonged or severe headaches, cranial neuropathies, and signs of elevated intracranial pressure in the setting of a history concerning for an episode of AOM that may have gone untreated. Timely diagnosis and intervention of complicated otogenic infections are necessary to prevent permanent neurologic deficits and improve health outcomes in the pediatric population.

## Author Contributions

Merrick J. Harris is the Corresponding Author. Merrick J. Harris, Deepa Mukundan, and Janet Moore were involved in the patient’s care. Deepa Mukundan and Janet Moore oversaw the patient’s management. Merrick J. Harris and Paola Pedraza Cruz wrote the manuscript.

## Funding

The authors declare that there is no funding related to the publication of this article.

## Disclosure

All authors have reviewed and approved the final version.

## Consent

All the patients allowed personal data processing, and informed consent was obtained from all individual participants included in the study.

## Conflicts of Interest

The authors declare no conflicts of interest.

## Data Availability

Data sharing is not applicable to this article as no datasets were generated or analyzed during the current study.
